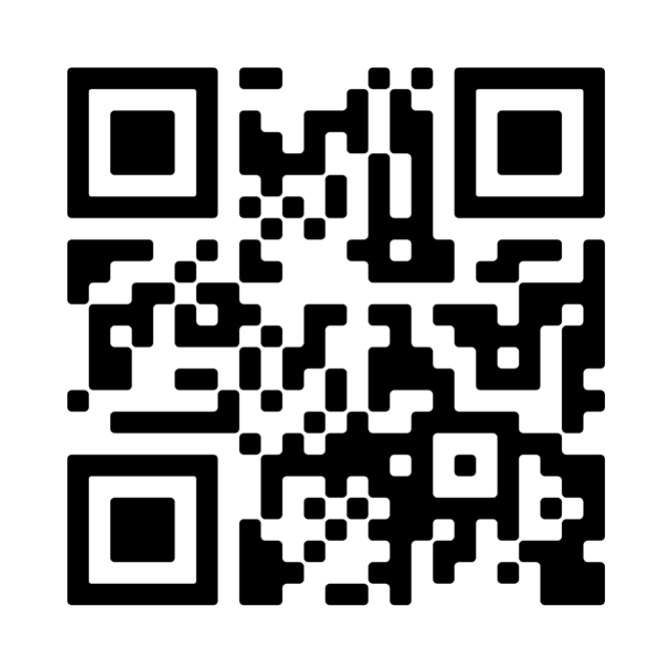# Laparoscopic Posterior Vaginal Plication plus Sacral Colpopexy for Severe Posterior Vaginal Prolapse: a Step-by-Step Video- Article

**DOI:** 10.52054/FVVO.15.4.103

**Published:** 2023-12-13

**Authors:** A Morciano, G Scambia, A ATinelli, G Marzo

**Affiliations:** Panico Pelvic Floor Center; Department of Gynaecology and Obstetrics, Pia Fondazione “Card. G. Panico”, Tricase, Lecce, Italy; Department of Gynaecology and Obstetrics, Fondazione Policlinico Universitario “A. Gemelli”, Università Cattolica del Sacro Cuore, Roma, Italy; Department of Gynaecology and Obstetrics” Veris Delli Ponti” Hospital, Scorrano, Lecce, Italy

**Keywords:** Posterior vaginal prolapse, laparoscopic sacral colpopexy, laparoscopic posterior plication

## Abstract

**Background:**

In 2023, our Centre validated a surgical approach for patients with anterior/apical prolapse associated with severe posterior colpocele, using a laparoscopic posterior vaginal plication (LPP) combined with standard sacral colpopexy (LSC), demonstrating significant benefits in terms of anatomical repair.

**Objectives:**

A step-by-step video demonstration of Laparoscopic Posterior Vaginal Plication (LPP) combined with “two-mesh” Sacral Colpopexy (LSC).

**Materials and Methods:**

Surgical technique of a LSC with 2 separate meshes is described.

**Results:**

This video-article describes, with a step-by-step approach, a combined prosthetic and fascial laparoscopic technique to treat severe posterior colpocele.

**Conclusions:**

LPP can be considered a feasible procedure during a standard LSC in patients with concomitant severe posterior prolapse.

## Learning Objective

To show a step-by-step demonstration of a surgical approach to pelvic organ prolapse (POP) patients with concomitant severe posterior colpocele, using a laparoscopic posterior vaginal fascial plication (LPP) (Morciano et al., 2023) combined with standard sacral colpopexy.

## Introduction

Laparoscopic sacral colpopexy (LSC) is the gold standard approach for anterior/apical pelvic organ prolapse (POP) and, when compared with vaginal procedures for apical suspension, it was linked with a significative reduction in anatomic recurrence or need to repeat surgery at 2 years (Maher et al., 2016).

Regarding posterior prolapse however, literature affirms that vaginal fascial native tissue repair seems to be preferable in terms of recurrence risk (Maher et al., 2013). Several authors highlighted, while LSC is considered a procedure that predominantly restores apical anatomy, a significative reduction in genital hiatus (GH) size and an amelioration in terms of posterior vaginal prolapse (Carter- Brooks et al., 2019; Fox and Staton, 2000). Recent studies proposed, in addition, a laparoscopic approach to anterior or posterior native tissue repair combined with apical sacral colpopexy or pectopexy suspension (Noé et al., 2019). In the light of these observations, in 2023 we validated a surgical approach to patients with anterior/ apical prolapse associated with severe posterior colpocele, using a Laparoscopic Posterior Vaginal Plication (LPP) combined with a standard LSC, demonstrating significant beneficial results in terms of anatomical repair (Morciano et al., 2023). This video-article is a step-by-step video demonstration of this combined fascial/prosthetic laparoscopic technique.

## Patients and methods

A 68-year-old woman, gravida 2 para 2, with no other surgical history, was referred to our pelvic centre with a diagnosis of symptomatic anterior and apical pelvic prolapse and a concomitant severe posterior vaginal prolapse (POP-Q scores: Aa +3.0, Ba +5.0, C +8.0, GH 6.0, pb 1.0, TVL 10, Ap +3.0, Bp +5.0, D +5.0) (Bump et al., 1996). A Laparoscopic Supracervical Hysterectomy (LScH), due to uterine fibromatosis was undertaken followed by a vesico-vaginal and recto-vaginal dissection (Lizee et al., 2017).

The surgical procedure of our combined LPP consisted of the following steps:

1. Posterior recto-vaginal dissection: it is carried out lateral to the rectum upward to identify the tendinous centre of perineum and the pelvic parietal fascia covering the levator ani muscle.
2. Posterior mesh fixation (caudal sutures): the caudal part of the mesh (Restorelle XL, Coloplast Corp., Minneapolis, MN, USA) was fixed laterally at the iliococcygeus muscle aponeurosis and medially at the caudal part of vagina.
3. Posterior vaginal plication: it is practiced using 4 extracorporeal interrupted 0 delayed absorbable polydioxanone monofilament sutures (PDO 0, RESORBA Medical GmbH, Nürnberg, Deutschland), placed horizontally, in small steps and in a semicircle and horizontal manner, duplicating the entire vaginal length.
4. Posterior mesh fixation (cranial sutures): the mesh is cranially tension-free fixed to the uterine insertions of uterosacral ligaments.

After anterior mesh positioning (Campagna et al., 2018), vaginal suspension was obtained by mesh fixation to the longitudinal vertebral ligament with laparoscopic tacks (CapSure™ Permanent Fixation System; Bard, Franklin Lakes, USA). A written informed consent was obtained from the patient for surgery and case presentation.

## Results

The procedure was performed without complications and patient was discharged on postoperative day 2. No de novo dyspareunia and defecatory dysfunctions were observed. At 1 year follow-up, we observed absence of the anterior-apical and posterior prolapse. The 12-months follow-up POP-Q scores were: Aa -2.6, Ba -2.4, C -6.0, GH 1.9, pb 2.0, TVL 10, Ap -2.3, Bp -2.4, D -5.0. In particular, Ap and GH points of POP-Q (Bump et al., 1996) were considerably improved (-2.3 and 1.9, respectively). The Patient Global Impression of Improvement (PGI-I) (Srikrishna et al., 2010) was “very much better”.

## Discussion

This video-article shows, with a step-by-step approach, a combined prosthetic and fascial laparoscopic technique for severe posterior colpocele combined with anterior/apical prolapse.

This surgical approach, unlike previous studies on laparoscopic anterior single-mesh sacral colpopexy or pectopexy suspension and combined native tissue repair, specifically analysing apical anatomy (Noé et al., 2019), was expressly validated for anterior/ apical prolapse associated with severe posterior colpocele, demonstrating significant results in terms of anatomical posterior repair (Morciano et al., 2023).

Several considerations should be made regarding our specific surgical procedure. Supracervical hysterectomy during sacral colpopexy is not a mandatory procedure. The literature showed no anatomical differences between hysterectomy and the conservative approach; both procedures are safe and effective POP treatments (Arcieri et al., 2023). Patients who no longer desire uterine preservation could be encouraged to consider LScH. Conservative LSC is an alternative in women who are strongly motivated to preserve their uterus in the absence of abnormal uterine findings (Arcieri et al., 2023). In this video procedure, LScH was practiced in accordance with patient’s will.

In addition, for isolated posterior compartment literature suggest a vaginal fascial approach (Maher et al., 2013). We studied this laparoscopic combined technique, in particular, only for patients with apical/anterior prolapse and a concomitant severe posterior colpocele. In patients with isolated colpocele we practice, as guidelines advise, native- tissue rectocele repair.

As several authors highlighted (Gadonneix et al., 2004; Lizee et al., 2017), during LSC, the “two meshes technique” is the most appropriate, because the verticalisation of the vaginal axis, caused by the anterior mesh traction, opens the Douglas cul de sac. A simultaneous mesh on the posterior compartment is, then, necessary to prevent the risk of posterior colpocele and to reinforce the rectovaginal closure. The strength of this procedure is precisely in matching, in patients with medium-severe anterior- apical and simultaneous severe posterior pelvic prolapse, the benefits of “two-meshes” sacral colpopexy (for anterior/apical prolapse) and of native tissue repair (for severe posterior prolapse), in a single laparoscopic approach.

## Conclusions

LPP can be considered a feasible procedure during a standard LSC in patients with concomitant severe posterior prolapse.

## Video scan (read QR)

https://vimeo.com/855801049/75efcb8068?share=copy

**Figure:**